# Vitamins improve the effect of heavy metal exposure in arthritis after hysterectomy

**DOI:** 10.3389/fnut.2025.1623490

**Published:** 2025-08-04

**Authors:** Binkai Xu, Xian Wu, Zhiwei Liu, Bin Yu

**Affiliations:** Changzhou Maternal and Child Health Care Hospital, Changzhou Medical Center of Nanjing Medical University, Changzhou, China

**Keywords:** hysterectomy, arthritis, heavy metal, lead, vitamin

## Abstract

**Background:**

The interplay between gynaecological surgeries and arthritis pathogenesis remains poorly understood. This study offers new insights into potential health risks associated with post-hysterectomy.

**Methods:**

The cross-sectional study utilized data from the National Health and Nutrition Examination Survey (NHANES) from 2007 to 2018, which cannot establish the causation. The effects of five serum heavy metal and nine vitamin intakes were evaluated.

**Results:**

A total of 3,121 participants with complete data from NHANES (2007 ~ 2018) were included in this study. The prevalence of arthritis among participants having undergone hysterectomy was significantly increased (58.25% vs. 31.64%, *p* < 0.001). Meanwhile, the levels of blood lead were significantly increased in women having undergone gynaecological surgery (*p* < 0.001) and women with arthritis (*p* < 0.001). After additionally adjusting, hysterectomy was still associated with an increased risk of arthritis (OR = 3.33, *p* < 0.0001). A non-linear (L-shaped) relationship was observed in blood lead, mercury, and cadmium (*p* for non-linearity <0.001). Blood lead was the highest weighted quantile sum (WQS) weigh among five heavy metals, with the highest contributions of 0.72. Mediation analysis demonstrated that blood lead accounted for 6.02% of the observed association between hysterectomy and arthritis (*p* < 0.001). The RCS curves confirmed that there was a non-linear (L-shaped) relationship between vitamin K, vitamin D, and the risk of arthritis caused by hysterectomy (*p* < 0.001).

**Conclusion:**

Hysterectomy is associated with an increased risk of arthritis, with a focus on blood lead as a mediating factor and vitamin intake as a potential protective factor. It will contribute to the long-term health management after hysterectomy.

## Highlighting

- The first large-scale epidemiological investigation systematically evaluated the relationship between hysterectomy and arthritis risk in women, with a focus on elucidating the mediating role of serum heavy metals (particularly blood lead) and the protective effects of dietary micronutrients (notably vitamin K and vitamin D).
- Hysterectomy, especially when combined with oophorectomy, is independently associated with a 3.3 ~ 4.7-fold increased risk of arthritis in women.
- Elevated blood lead levels mediate approximately 6% of this association, exhibiting a non-linear threshold effect.
- Higher dietary intake of vitamin K and vitamin D significantly attenuates arthritis risk in hysterectomized women, with protective thresholds identified at 212.0mcg/day and 10.8 mcg/day, respectively.

## Introduction

As the most common gynecologic operation, approximately 600,000 hysterectomies are performed annually in the United States for common gynaecologic benign indications, such as uterine fibroids, abnormal uterine bleeding, endometriosis, and pelvic organ prolapse (1). Although its prevalence has declined recently, hysterectomy continues to carry significant long-term health implications (2–4). Recent studies have suggested that there were associations between hysterectomy and increased risks of cardiovascular disease (5), stroke (6), metabolic disorders (7), kidney stone disease (8), and osteoporosis (9).

Arthritis, encompassing osteoarthritis (OA), rheumatoid arthritis (RA), and related inflammatory joint diseases, disproportionately affects women (10). Hormonal fluctuations, genetic predisposition, obesity, and aging are established contributors. Postmenopausal women, in particular, face elevated risks due to estrogen depletion, which exacerbates joint inflammation and cartilage degradation (11). Beyond hormonal factors, environmental exposures, including heavy metals, and nutritional deficiencies have recently emerged as modifiable risk factors (12, 13). However, the interplay between gynaecological surgeries and arthritis pathogenesis remains poorly understood.

Bone health of women after hysterectomy is a brand-new study field. Its potential role in arthritis remains underexplored. Relatively, it is clear at present that hysterectomy is related to female osteoporosis. Seo and Yuk (9), Choi et al. (14), and Xu et al. (15) reported that the risk of osteoporosis was increased in women who had undergone hysterectomy regardless of bilateral oophorectomy status. The adjusted odds ratios (ORs) were 1.28 ~ 1.84. However, whether it also increases the risk of fracture was controversial (9, 15). In relation to arthritis, studies are even rarer. In J Stöve’s group, 86.2% patients after hysterectomy developed bilateral OA and 33.3% occurred generalized OA (16). Recently, a Taiwanese cohort study further identified hysterectomy as an independent risk factor for osteoarthritis, especially knee OA (adjusted OR = 1.25, 95%CI = 1.13–1.38) (17). They speculated that it might be related to estrogen deficiency. No further research has been carried out.

In recent years, the role of heavy metals in the occurrence and the development of arthritis have attracted much attention, with primary focus on arsenic, cadmium, zinc, and lead (18, 19). It is generally acknowledged that the high exposure of heavy metals may be a synergistic risk factor associated with arthritis. Interestingly, the intake of some micronutrients may reverse such damage (20, 21). However, there is still a lack of large cohort clinical studies. Meanwhile, hysterectomy may indirectly influence these metals. For instance, estrogen deficiency post-surgery could impair heavy metal detoxification pathways, increasing their long-term exposure (22, 23). In addition, heavy metals may also acts as potent xenoestrogens with the risk to human health (24). In summary, the relationship among hysterectomy, arthritis, heavy metals, and micronutrients is highly complicated.

In order to explore this scientific problem, from the National Health and Nutrition Examination Survey (NHANES) (25, 26), we investigated the relationship between hysterectomy and arthritis and explored the role of heavy metals and the protective effect of vitamins. This study offers new insights into potential health risks associated with post-hysterectomy and hope to contribute to improving public health outcomes.

## Materials and methods

### Data source and study population

**Figure 1:**
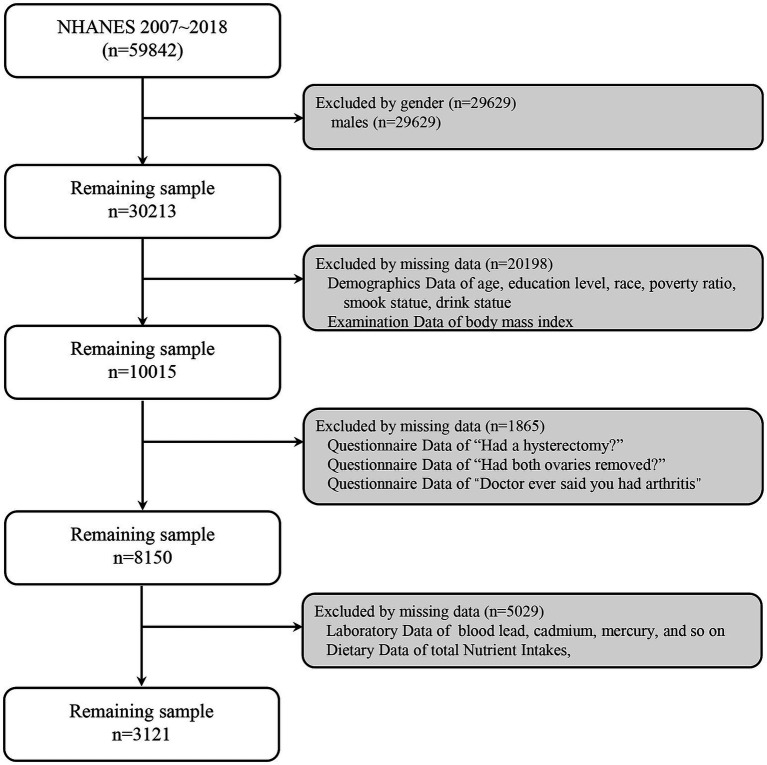
Flowchart of participant selection from the NHANES 2007–2018.

The cross-sectional study was from the NHANES, and the cross-sectional design limits the ability to establish causality. The participant’s selection is illustrated in Figure 1. Of 59,842 initial subjects from the NHANES (2007–2018), 29,629 were excluded due to gender. A total of 20,198 were excluded due to incomplete data on age, education level, race, poverty ratio, smoking status, drink statue, and body mass index (BMI). A total of 1,865 were excluded due to missing data on hysterectomy and the data on arthritis. A total of 5,029 were excluded due to missing data on blood heavy metals and total nutrient intakes. Finally, 3,121 women were included in this study. According to whether they had undergone hysterectomy and/or oophorectomy, the subjects were divided into four groups: G0 (*n* = 2,298, normal control), G1 (*n* = 388, only having undergone hysterectomy), G2 (*n* = 13, only having undergone oophorectomy), and G3 (*n* = 422, having undergone hysterectomy and oophorectomy).

### Definition of hysterectomy and/or oophorectomy

Similar to the previous study (8), hysterectomy was measured by the following question: “Had a hysterectomy? (RHD280).” Women who answered “yes” were considered having hysterectomy. Similarly, ovaries removed were defined according to the question: “Had both ovaries removed? (RHD305).” Women who answer “yes” were considered to have the oophorectomy.

### Definition of arthritis

Similar to the previous study (27), arthritis was measured with the following question (MCQ160a): Has a doctor or other health professional ever told you that you had arthritis? Women who answered yes were considered to have arthritis.

### Measurements of blood heavy metals

The levels of blood heavy metals were obtained from the lead, cadmium, total mercury, selenium and manganese—blood (PBCD) dataset of laboratory data, including measurements of blood lead (umol/L), blood cadmium (umol/L), blood mercury (umol/L), blood selenium (umol/L), and blood manganese (umol/L).

### Measurements of total nutrient intake

Related dietary parameters were derived from the Dietary Interview—Total Nutrient Intakes, First Day (DR1TOT). In this study, we focused on vitamin intake, including the following: vitamin A (mcg), vitamin B1 (mg), vitamin B2 (mg), vitamin B6 (mg), vitamin B12 (mcg), vitamin C (mg), vitamin D (D2 + D3) (mcg), vitamin E (mg), and vitamin K (mcg).

### Other covariates

Similar to several previous studies (28, 29), age, gender, race education level, race, and poverty ratio were obtained from demographics data. BMI and weight were collected from examination data. Smoking status and drink status were collected during in-home interviews.

### Statistical analysis

DecisionLinnc1.0 software (30) was used for data analysis, which is a platform that integrates multiple programming language environments. The logistic regression analysis model was used across three distinct models to examine the relationship between hysterectomy and arthritis. Subgroup analyses were also conducted. Next, restricted cubic splines (RCS) were utilized to explore potential non-linear relationships between blood heavy metals, vitamin, and the risk of arthritis caused by hysterectomy. The parallel mediation analysis was performed to clarify their intermediary roles. Weighted quantile sum (WQS) regression was used to explore the overall effect of metals on arthritis. A *p*-value of < 0.05 was considered statistically significant.

## Results

### Baseline participant characteristics

A total of 3,121 participants with complete data from NHANES (2007 ~ 2018) were included in this study. Table 1 presents the baseline characteristics of the participants according to hysterectomy. Of 3,121 participants, 1,237 (39.63%) women had arthritis. Compared to the G0 group, the rates of arthritis in the participants having undergone hysterectomy (G1) were significantly increased (58.25% vs. 31.64%, *p* < 0.001). When women who underwent hysterectomy and had their ovaries removed at the same time (G3), her arthritis rate further increased to 65.64%. In addition, among women who had undergone gynecological surgery were generally older (*p* < 0.001) and had a higher BMI (*p* < 0.001), but the smoking rate was significantly reduced (*p* = 0.001).

**Table 1:** Baseline participant characteristics according to hysterectomy.

Variable names	Overall	Operation mode				p-value
		G0	G1	G2^#^	G3	
*n*	3,121	2,298	388	13	422	
Age (year)	49.11 ± 15.98	45.75 ± 15.65	57.40 ± 12.49	58.81 ± 11.56	60.83 ± 11.88	<0.001
20 ~ 34	649 (20.79%)	638 (27.76%)	4 (1.03%)	1 (7.69%)	6 (1.42%)	
35 ~ 44	529 (16.95%)	450 (19.58%)	49 (12.63%)	1 (7.69%)	29 (6.87%)	
45 ~ 55	650 (20.83%)	468 (20.37%)	97 (25.00%)	2 (15.38%)	83 (19.67%)	
> =55	1,293 (41.43%)	742 (32.29%)	238 (61.34%)	9 (69.23%)	304 (72.04%)	
Race (%)						0.039
Mexican American	307 (9.84%)	246 (10.70%)	33 (8.51%)	0 (0.00%)	28 (6.64%)	
Other Hispanic	260 (8.33%)	206 (8.96%)	34 (8.76%)	1 (7.69%)	19 (4.50%)	
Non-Hispanic White	1,801 (57.71%)	1,306 (56.83%)	217 (55.93%)	7 (53.85%)	271 (64.22%)	
Non-Hispanic Black	594 (19.03%)	413 (17.97%)	92 (23.71%)	2 (15.38%)	87 (20.62%)	
Other race	159 (5.09%)	127 (5.53%)	12 (3.09%)	3 (23.08%)	17 (4.03%)	
Poverty income ratio	2.74 ± 1.66	2.72 ± 1.68	2.77 ± 1.61	3.61 ± 1.57	2.84 ± 1.58	0.081
< 5.0	2,681 (85.90%)	1,967 (85.60%)	337 (86.86%)	10 (76.92%)	367 (86.97%)	
> =5.0	440 (14.10%)	331 (14.40%)	51 (13.14%)	3 (23.08%)	55 (13.03%)	
Education level (%)						0.124
Less than 9th grade	225 (7.21%)	149 (6.48%)	32 (8.25%)	0 (0.00%)	44 (10.43%)	
9–11th grade	592 (18.97%)	434 (18.89%)	89 (22.94%)	3 (23.08%)	66 (15.64%)	
High school graduate	777 (24.90%)	572 (24.89%)	91 (23.45%)	2 (15.38%)	112 (26.54%)	
Some college	1,028 (32.94%)	748 (32.55%)	131 (33.76%)	5 (38.46%)	144 (34.12%)	
College graduate or above	499 (15.99%)	395 (17.19%)	45 (11.60%)	3 (23.08%)	56 (13.27%)	
BMI (kg/m^2^)	29.17 ± 7.38	28.88 ± 7.46	30.08 ± 7.12	27.69 ± 6.57	30.10 ± 7.04	<0.001
Normal weight (%)	895 (28.68%)	714 (31.07%)	91 (23.45%)	5 (38.46%)	85 (20.14%)	
Overweight (%)	882 (28.26%)	629 (27.37%)	106 (27.32%)	1 (7.69%)	146 (34.60%)	
Obesity (%)	1,344 (43.06%)	955 (41.56%)	191 (49.23%)	7 (53.85%)	191 (45.26%)	
Smoking statue						0.001
No	1,613 (51.68%)	1,098 (47.78%)	233 (60.05%)	7 (53.85%)	275 (65.17%)	
Yes	1,508 (48.32%)	1,200 (52.22%)	155 (39.95%)	6 (46.15%)	147 (34.83%)	
Drinking statue						0.076
No	658 (21.08%)	452 (19.67%)	94 (24.23%)	2 (15.38%)	110 (26.07%)	
Yes	2,463 (78.92%)	1,846 (80.33%)	294 (75.77%)	11 (84.62%)	312 (73.93%)	
Arthritis						<0.001
No	1,884 (60.37%)	1,571 (68.36%)	162 (41.75%)	6 (46.15%)	145 (34.36%)	
Yes	1,237 (39.63%)	727 (31.64%)	226 (58.25%)	7 (53.85%)	277 (65.64%)	

^#^The G2 subgroup (ovariectomy without hysterectomy) includes only *n* = 13, limiting statistical power.

Table 2 compares the levels of blood heavy metals according to hysterectomy or arthritis, respectively. Notably, the levels of blood lead were significantly increased in women having undergone gynecological surgery (*p* < 0.001). At the same time, the blood lead levels of women with arthritis were also significantly increased (*p* < 0.001).

**Table 2:** Comparison of blood heavy metals according to hysterectomy and arthritis.

	Hysterectomy					Arthritis		
	G0	G1	G2^#^	G3	*p*-value	No	Yes	*p*-value
Blood lead (umol/L)	0.06 ± 0.04	0.08 ± 0.08	0.08 ± 0.04	0.08 ± 0.05	<0.001	0.06 ± 0.04	0.08 ± 0.06	<0.001
Blood mercury (nmol/L)	6.78 ± 9.17	6.29 ± 6.94	7.84 ± 6.09	6.12 ± 6.71	0.306	6.89 ± 9.23	6.22 ± 7.50	0.201
Blood cadmium (nmol/L)	7.43 ± 7.82	6.99 ± 6.13	5.89 ± 4.94	6.79 ± 5.84	0.749	7.12 ± 7.18	7.59 ± 7.76	0.039
Blood selenium (umol/L)	2.48 ± 0.45	2.44 ± 0.57	2.31 ± 0.21	2.46 ± 0.34	0.001	2.49 ± 0.47	2.44 ± 0.42	0.274
Blood manganese (nmol/L)	186.99 ± 71.46	165.82 ± 54.06	177.71 ± 104.66	181.97 ± 85.09	0.007	184.17 ± 69.86	183.71 ± 75.90	0.799

^#^The G2 subgroup (ovariectomy without hysterectomy) includes only *n* = 13, limiting statistical power.

### Associations between hysterectomy and arthritis

We have established three models before and after adjusting for confounding factors. As shown in Table 3, these three models established a statistically significant association between hysterectomy and arthritis. In the unadjusted model (Model 1), the risk of arthritis for women who had undergone hysterectomy was significantly increased. The odds ratio (OR) and 95% confidence intervals (CIs) were 3.55 (2.49, 5.04) (*p* < 0.0001). After additionally adjusting for general data confounding factors such as race, education, poverty–income ratio (Model 2), hysterectomy was still associated with an increased risk of arthritis (OR = 3.47, *p* < 0.0001). Furthermore, Model 3 still showed this trend of increased risk after additionally adjusting for BMI, smoking status, and drinking status (OR = 3.33, *p* < 0.0001). The risk would further increase when woman underwent hysterectomy and oophorectomy. The ORs were 5.08, 4.94, and 4.67, respectively (*p* < 0.0001).

**Table 3:** Multivariable-adjust ORs and 95%CI of hysterectomy and arthritis.

Hysterectomy	Model 1		Model 2		Model 3	
	OR (95%CI)	*p*-value	OR (95%CI)	*p*-value	OR (95%CI)	*p*-value
Overall (*n* = 3,121)						
G0	Reference		Reference		Reference	
G1	3.55 (2.49, 5.04)	<0.0001	3.47 (2.46, 4.90)	<0.0001	3.33 (2.36, 4.70)	<0.0001
G2^#^	2.08 (0.53, 8.21)	0.2917	2.12 (0.52, 8.55)	0.2854	2.23 (0.59, 8.41)	0.2337
G3	5.08 (3.76, 6.86)	<0.0001	4.94 (3.63, 6.72)	<0.0001	4.67 (3.41, 6.39)	<0.0001

Model 1 without adjustments. Model 2 was additionally adjusted for race, education level, and poverty. Model 3 was additionally adjusted for BMI, smoking status, and drink statue. ^#^The G2 subgroup (ovariectomy without hysterectomy) includes only *n* = 13, limiting statistical power.

**Figure 2:**
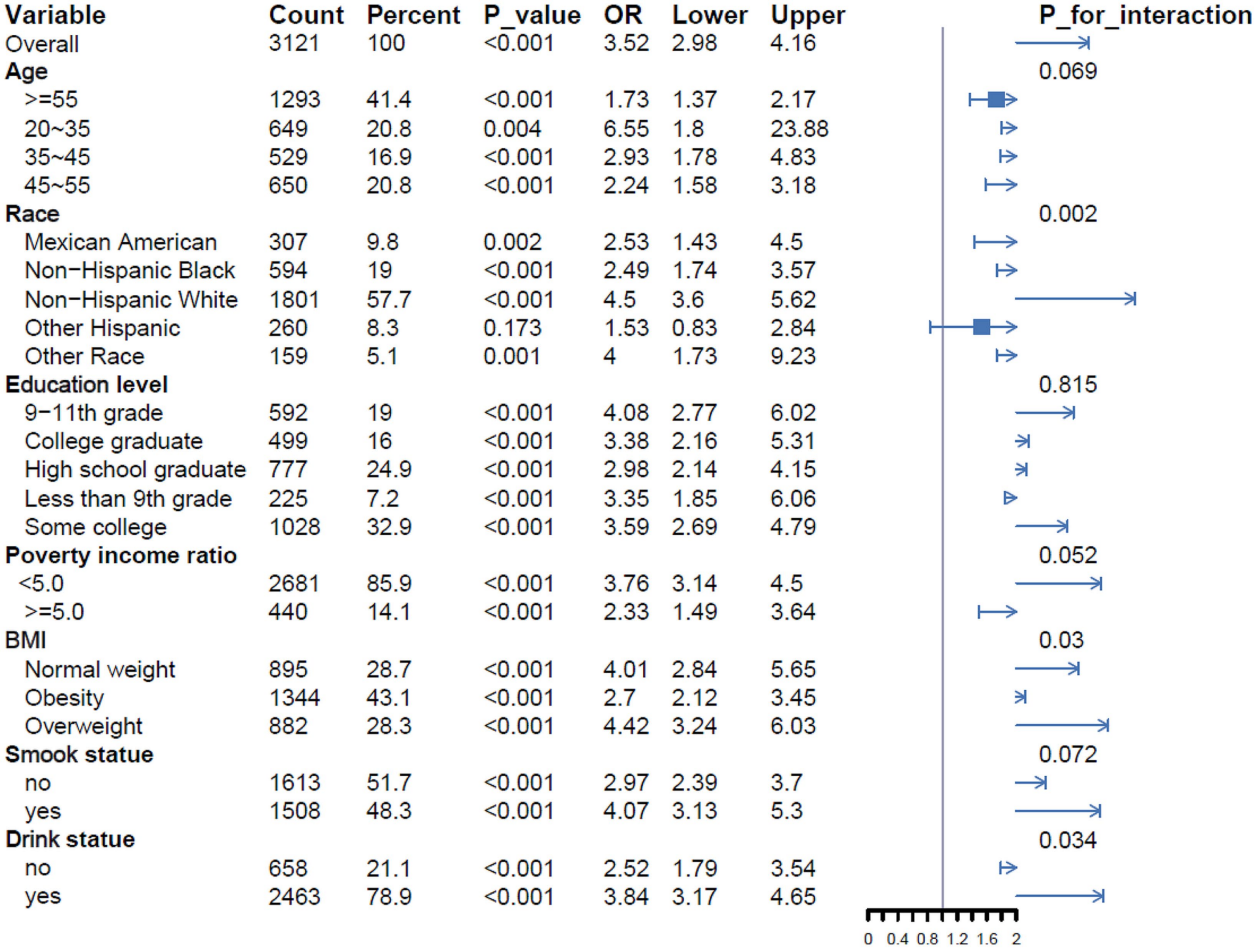
Subgroup analysis of the associations between hysterectomy and arthritis.

To examine potential differences in the relationship between hysterectomy and arthritis in specific populations, we conducted subgroup analyses and interaction tests by age, race, education level, poverty ratio, BMI, smoking status, and drinking status. As shown in Figure 2, significant interactions were found between race, BMI, and drink status.

### Effects of blood heavy metals

**Figure 3:**
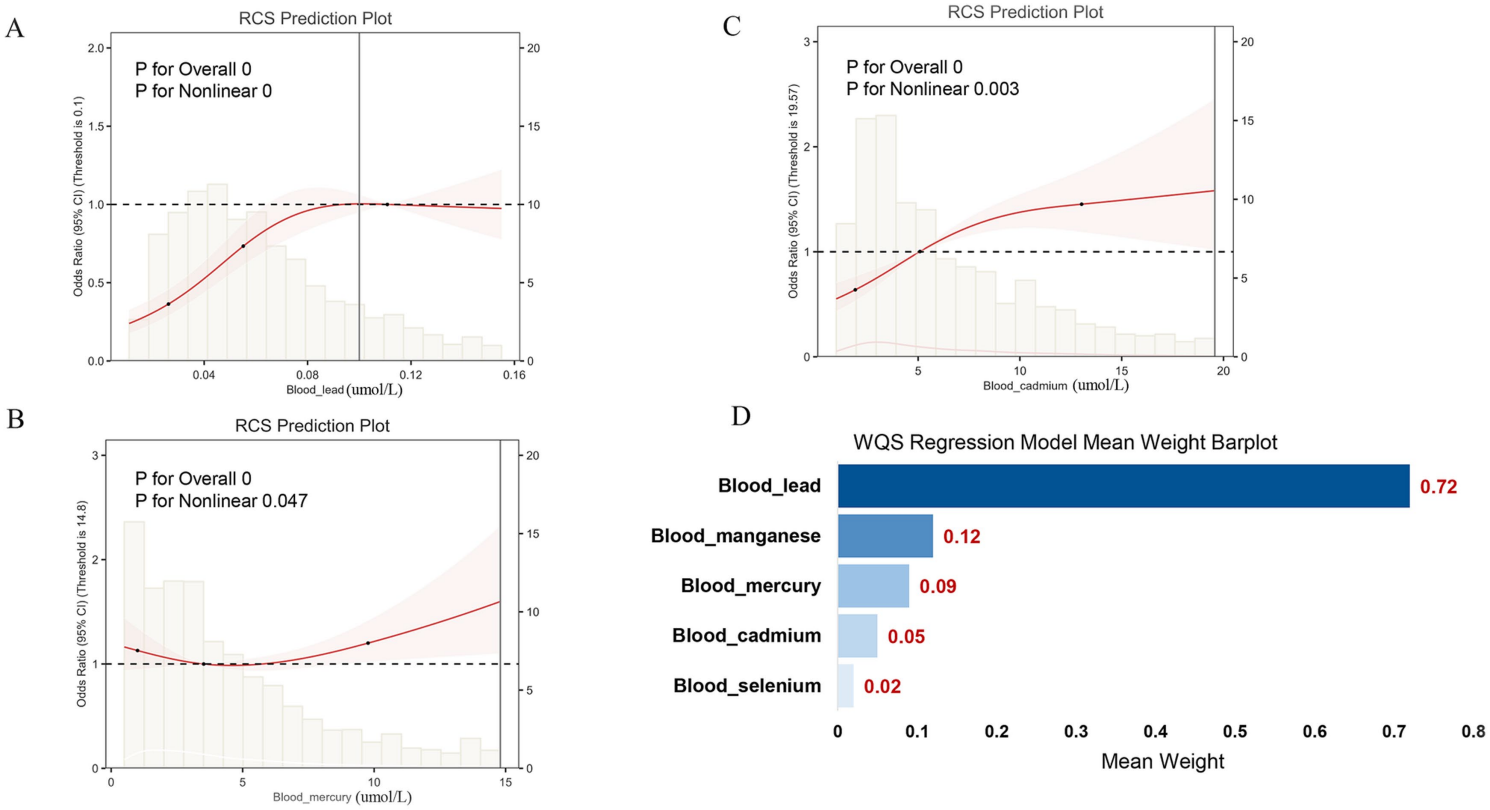
Association between blood heavy metals and the risk of arthritis caused by hysterectomy. **(A)** Restricted cubic splines curves of lead. **(B)** Restricted cubic splines curves of mercury. **(C)** Restricted cubic splines curves of cadmium. **(D)** WQS regression model of blood heavy metals. Results were adjusted for race, education level, smoking status, drink statue, and BMI.

**Figure 4:**
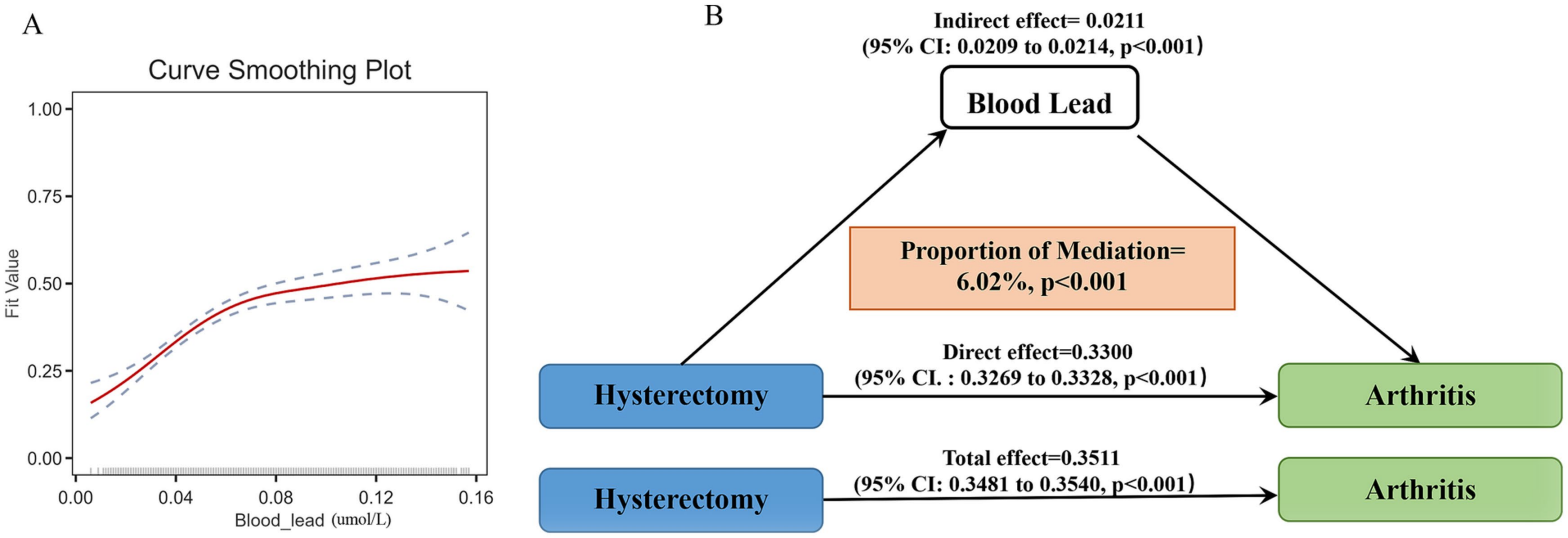
Mediation of blood lead in the association between hysterectomy and arthritis. **(A)**. Smooth curve fitting of blood lead. **(B)**. Mediation analysis of blood lead. Results were adjusted for race, education level, smoking status, drink statue, and BMI.

First, the RCS curves were employed to display the association between the levels of blood heavy metals and the risk of arthritis caused by hysterectomy. After adjusting for multiple variables, evidence of a non-linear (L-shaped) relationship were observed in blood lead, mercury, and cadmium (*p* for non-linearity <0.001) (Figures 3A–C). However, there was no non-linear (L-shaped) relationship between blood selenium, manganese, and the odds ratio (Supplementary Figure 1). Second, WQS regression models were used to evaluate the impact of blood heavy metals on the risk of arthritis caused by hysterectomy. Among the five heavy metals, blood lead was the highest WQS weight, with the highest contribution at 0.72 (Figure 3D). Third, smooth curve fitting was used to clarify the relationship between blood lead and arthritis risk (Figure 4A). An inflection point (0.10 μmol/L) was determined by threshold effect analysis. It was particularly noteworthy that each unit intake increase in blood lead amplified the risk of arthritis by 32% (*p* < 0.001) below this level. Next, to clarify whether blood lead plays an intermediary role between hysterectomy and arthritis, the parallel mediation analysis was carried out. Mediation analysis demonstrated that blood lead accounted for 6.02% of the observed association between hysterectomy and arthritis (*p* < 0.001, Figure 4B). No significant mediated effects were found on other heavy metals.

### Improvement of nutrient intake

**Figure 5:**
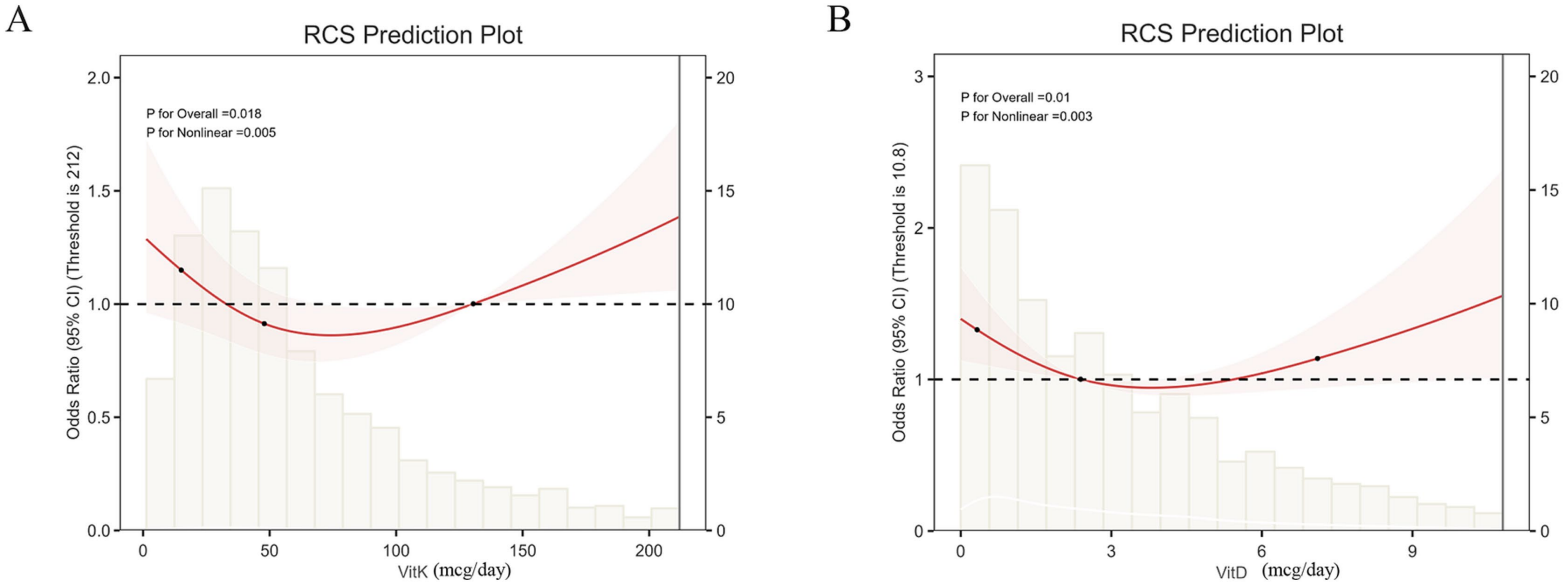
Dietary fiber intake reduces the risk of arthritis. **(A)**. Restricted cubic splines curves of vitamin K. **(B)**. Restricted cubic splines curves of vitamin D. Results were adjusted for gender, race, education level, smoking status, and BMI.

We evaluated the potential of nine vitamin intakes in reducing the risk of arthritis caused by hysterectomy. The RCS curves confirmed that there was a non-linear (L-shaped) relationship between vitamin K, vitamin D, and the odds ratio of the risk of arthritis caused by hysterectomy (*p* for non-linearity <0.001) (Figures 5A,B). Additionally, smooth curve fitting also clarified that arthritis after hysterectomy would be reduced with the intake of vitamin K and vitamin D (Supplementary Figure 2). With threshold effect analysis, both optimal inflection points were 212.0 mcg/day for vitamin K and 10.8 mcg/day for vitamin D, respectively. Beyond this level, each additional unit of intake would significantly reduce the risk of arthritis by 20 and 53%, respectively (*p* < 0.001).

## Discussion

The present study represents the first large-scale epidemiological investigation to systematically evaluate the relationship between hysterectomy and arthritis risk in women, with a focus on elucidating the mediating role of serum heavy metals (particularly blood lead) and the protective effects of dietary micronutrients (notably vitamin K and vitamin D). Leveraging data from the NHANES cohort (2007–2018), our findings revealed three key observations: (1) hysterectomy, especially when combined with oophorectomy, is independently associated with a 3.3 ~ 4.7-fold increased risk of arthritis in women; (2) elevated blood lead levels mediate approximately 6% of this association, exhibiting a non-linear threshold effect; and (3) higher dietary intake of vitamin K and vitamin D significantly attenuates arthritis risk in hysterectomized women, with protective thresholds identified at 16.7 mcg/day and 1.3 mcg/day, respectively. These results advance our understanding of the long-term musculoskeletal consequences of hysterectomy and highlight actionable targets for mitigating post-surgical morbidity.

Our analysis corroborates emerging evidence linking hysterectomy to arthritis morbidity. The adjusted odds ratio (OR = 3.33, 95%CI: 2.49–5.04) for arthritis in hysterectomized women aligns with prior reports, such as the Taiwanese cohort study identifying a 25% increased risk of knee osteoarthritis (OA) post-hysterectomy (adjusted OR = 1.25). Notably, the risk escalated further in women who underwent concurrent oophorectomy (OR = 4.67), underscoring the compounded effects of estrogen depletion. Mechanistically, the uterus is not merely a reproductive organ but an endocrine-active tissue contributing to extra-ovarian estrogen synthesis and immunomodulation. Its removal disrupts systemic estrogen signalling, which regulates cartilage homeostasis, synovial inflammation, and osteoclast activity (31). Estrogen deficiency post-hysterectomy may accelerate joint degeneration via upregulation of matrix metalloproteinases (MMPs) (32) and interleukin-1β (IL-1β) (33), pathways implicated in both OA and rheumatoid arthritis (RA) pathogenesis. Furthermore, surgical trauma and postoperative inflammation could induce epigenetic modifications in joint tissues, priming them for accelerated aging.

A groundbreaking finding of this study is the identification of blood lead as a partial mediator (6.02%, *p* < 0.001) of the hysterectomy-arthritis relationship. Women with hysterectomy exhibited significantly higher levels of blood lead. Blood lead exposure amplified arthritis risk disproportionately, suggesting that even subclinical elevations may synergize with post-surgical metabolic shifts to harm joint health. Lead’s role in arthritis pathogenesis is well documented. Our mediation analysis suggested that hysterectomy exacerbated lead retention and further led to the occurrence and development of arthritis. However, the exact reason is still unclear. Studies have found that the expression of metallothionein I (MT-I) shows estrogen-dependent characteristics (34), while metallothionein has a broad potential for application in alleviating environmental heavy metal pollution (35). This might be one reason for the storage of blood lead due to estrogen deficiency after a hysterectomy. Interestingly, for blood lead, the inflection point at 0.10 μmol/L represents a toxicity threshold: below this level, each unit increase in lead amplified arthritis risk greatly. The threshold effect (0.10 μmol/L) corresponds to blood lead levels observed in populations with environmental or occupational exposure, highlighting the need for stricter monitoring in hysterectomized women. Public health interventions targeting lead reduction could mitigate arthritis risks in this vulnerable group, such as minimizing exposure to contaminated water, cosmetics, or ceramics.

Our study pioneers the exploration of dietary micronutrients as modifiers of hysterectomy-associated arthritis. Hysterectomy induces a dual deficiency state: estrogen loss impairs vitamin D activation, while an altered gut microbiota reduces vitamin bioavailability. Hysterectomized women may face additional barriers to adequate intake, such as post-surgical dietary restrictions or gut dysbiosis. The uterus and ovaries interact with the gut microbiota through estrogen-mediated modulation of intestinal permeability. Hysterectomy disrupts this axis, potentially impairing fat-soluble vitamin absorption (e.g., vitamins K and D). Dietary interventions emphasizing leafy greens (rich in vitamin K) and fortified dairy products (vitamin D sources) could counteract these deficits. Vitamin K emerged as a potent protective factor, with intake above 212.0 mcg/day reducing arthritis odds by 20% (*p* < 0.001). Vitamin D exhibited similar benefits with the optimal inflection points 212.0 mcg/day The protective threshold of vitamin K, particularly through matrix Gla-protein (MGP), is essential for bone health and may help prevent joint calcification and inflammation (36, 37). Additionally, hysterectomy may induce vitamin D deficiency through estrogen, thus indicating that even moderate intake confers benefits. The non-linear dose–response relationships suggest that optimal—rather than maximal—intake confers protection.

However, there are some limitations to the present study. The study’s cross-sectional design limits the ability to establish causality. We did not further classify arthritis, such as osteoarthritis and rheumatoid arthritis. There was the potential for selection bias due to exclusion of participants with missing data (>50% of the initial sample). We could not clarify the arthritis that occurred after uterine surgery due to a large number of missing data. We could only analyze the average ages of the two events that occurred, which were 40.85 (age when had hysterectomy) and 57.87 (age when told you had arthritis), respectively. The sample size of G2 (only having undergone ovaries removal) was too small, which might affect the analysis results of the subsequent group. However, in the results section, we focused on women who underwent hysterectomy (G1 and G3). The measurement of main variables (such as hysterectomy, arthritis, and dietary vitamin intake) relies on self-reported data, which is susceptible to recall bias. Additionally, despite adjusting for several potential confounders, the absence of important covariates (such as menopausal status, hormone replacement therapy, and time since surgery) could influence the results.

In conclusion, hysterectomy is associated with an increased risk of arthritis, with focus on blood lead as a mediating factor and vitamin intake as a potential protective factor. It will contribute to long-term health management after hysterectomy.

## Data Availability

The original contributions presented in the study are included in the article/Supplementary material, further inquiries can be directed to the corresponding authors.
