# Supplementary material for: Vitamins improve the effect of heavy metal exposure in arthritis after hysterectomy
Source: Front Nutr. 2025 Aug 4;12:1623490. doi: 10.3389/fnut.2025.1623490 (PMC12358258; doi:10.3389/fnut.2025.1623490)

Supplement Figure 1. Restricted cubic splines curves of blood selenium and manganese.

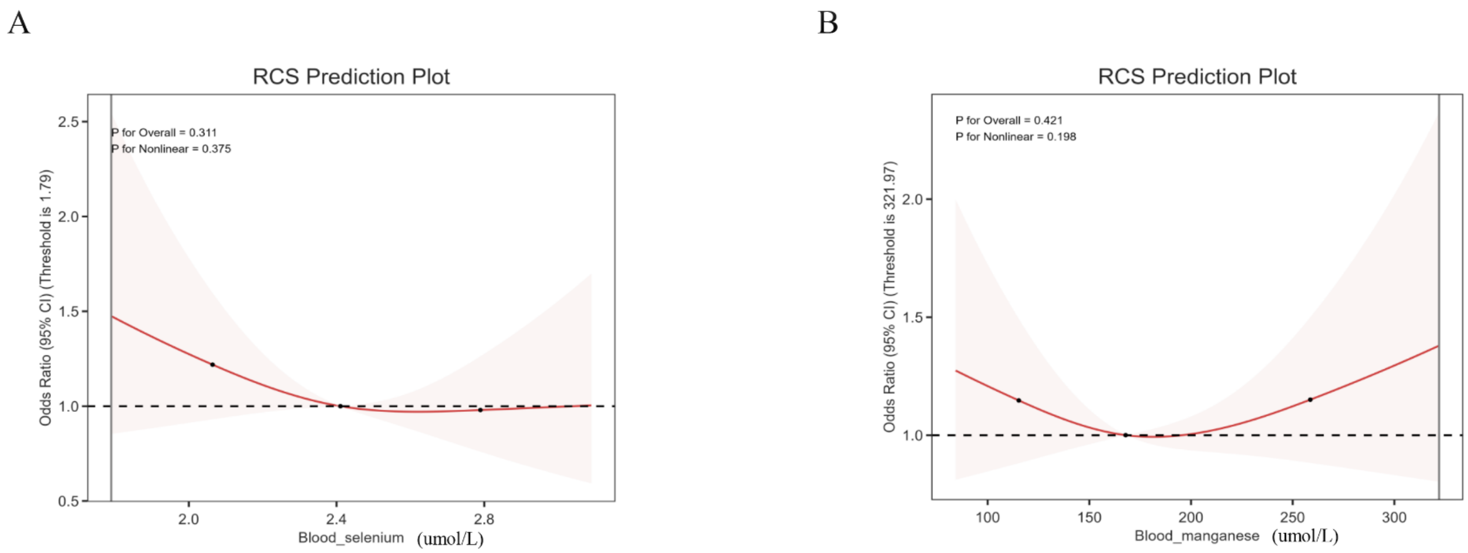

A. Restricted cubic splines curves of selenium

B. Restricted cubic splines curves of manganese

Supplement Figure 2. Smooth curve fitting of vitamin K and vitamin D

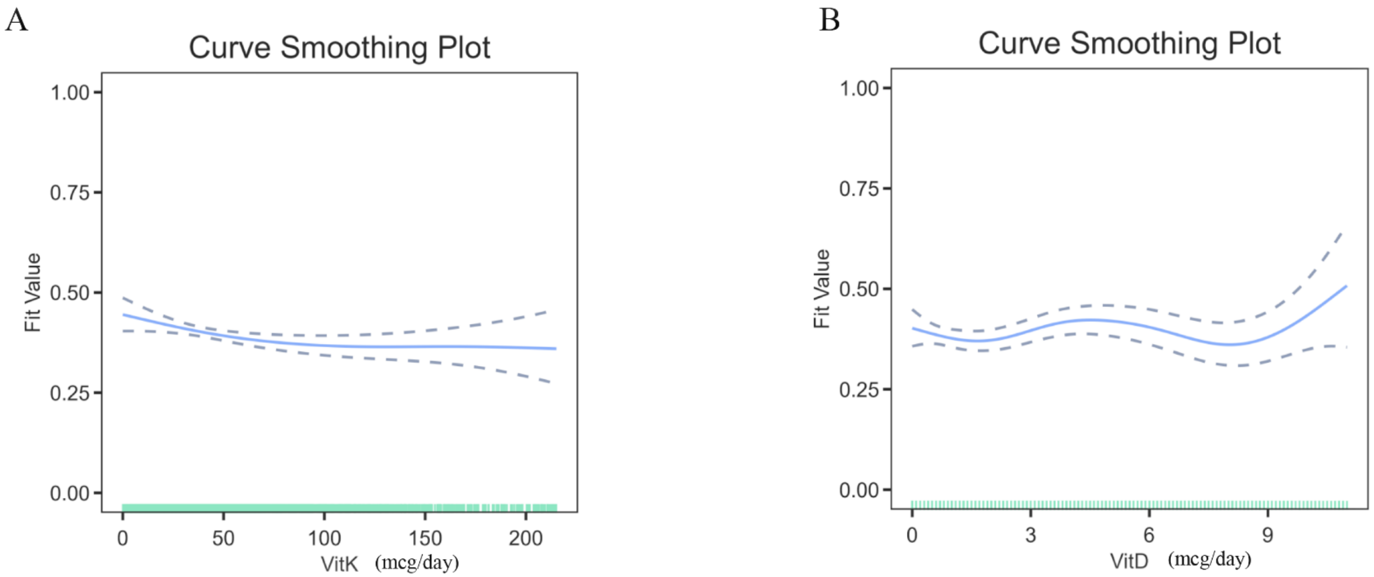

Supplement: Supplementary file 1 [file Image_1.pdf]
